# Individual and contextual determinants associated with traumatic dental injuries in children eight to ten years of age: a multilevel analysis

**DOI:** 10.2340/aos.v83.40891

**Published:** 2024-06-25

**Authors:** Veruska Medeiros Martins Bernardino, Larissa Chaves Morais de Lima, Érick Tássio Barbosa Neves, Saul Martins de Paiva, Ana Flávia Granville-Garcia

**Affiliations:** a Postgraduate Program in Dentistry, Dental School, State University of Paraíba – UEPB, Campina Grande, Paraiba, Brazil; b Department of Pediatric Dentistry and Orthodontics, Dental School, Federal University of Minas Gerais – UFMG, Belo Horizonte, Brazil

**Keywords:** Traumatic dental injury, electronic devices, schools

## Abstract

**Objective:**

Investigate individual and contextual determinants associated with traumatic dental injuries in schoolchildren.

**Methods:**

A cross-sectional study with 739 pairs of parents and children. Parents answered a sociodemographic questionnaire, the Family Adaptability and Cohesion Evaluation Scale and a questionnaire on the child’s use of electronic devices. Examinations of the children were conducted at the school by calibrated examiners using the diagnostic criteria proposed by Andreasen. Contextual variables of the school were also collected. Multilevel Poisson regression for complex samples was performed (*p* < 0.05).

**Results:**

The individual factors associated with the outcome were children of single parents (PR = 2.33; 95% CI: 1.79–2.66), practice of sports (PR = 2.46; 95% CI: 1.70–3.22), the daily use of electronic devices (PR = 1.78; 95% CI: 1.31–1.81), more than 2 h per day of screen time (PR = 3.84; 95% CI: 1.94–4.28) and chaotic family adaptability (PR = 4.22; 95% CI: 3.44–4.99). The contextual variables were studying at a public school (PR = 1.77; 95% CI: 1.02–3.05) and the presence of rigid floor in the school courtyard (PR = 1.99; 95% CI: 1.15–3.15).

**Conclusion:**

Individual determinants, studying at a public school and the presence of rigid floor in the school courtyard were associated with traumatic dental.

## Introduction

Traumatic dental injury (TDI) is a public health problem throughout the world because of the high prevalence and treatment costs [1]. Studies report that the prevalence of TDI in the permanent dentition ranges from 12.6% to 22%, and such injuries are more frequent in children 8 to 10 years of age [1, 2]. Factors that contribute to the occurrence of TDI include malocclusions, inadequate lip seal, overjet greater than 3 mm, sociodemographic factors, and obesity [1, 2].

The practice of sports can also be a frequent cause of injury in children because of the greater likelihood of falls and collisions with hard surfaces, playground equipment (swings and slides), and other children. In fact, less physical activity may be associated with less chance of having TDI. On the other hand, this highlights the importance of adopting protective measures to reduce the incidence of TDI in children while practicing sports [3]. The investigation of TDI in children who practice sports at school can provide important data for the adoption of prevention measures and health promotion strategies, such as the creation of safer environments for the practice of these activities in the school setting.

The increase in the use of electronic devices and the consequences of this habit for children has attracted the attention of researchers [4]. Time spent in front of a television, computer, tablet, or smartphone screen has been negatively associated with physical and cognitive development, with an increase in the likelihood of obesity, sleep problems, depression, and anxiety [3, 4]. Excessive exposure to the screen of electronic devices is associated with lower physical strength [5], which can exert a negative impact on balance, making children more prone to falls [6] and possible TDI. The conceptual hypothesis of this study is that the use of electronic devices and screen time of more than 2 h per day are associated with a greater frequency of TDI in children 8 to 10 years of age.

The family environment is an under-explored aspect of TDI. Family adaptability is related to the capacity for change in power relations among family members [7]. These interactions in the family environment affect the behavior of children [8, 9], as the authority structure in the family exerts an influence on parental supervision of the activities that children perform and this attitude may be a predictor of TDI.

The school context and physical structure of the school may also be associated with a greater frequency of TDI [10]. Physical aspects related to the school environment as a predictor of the occurrence of TDI in schoolchildren 8 to 10 years of age have been investigated little. Such knowledge could contribute to the creation of standardised norms regarding the physical structure of schools for the prevention of accidental injuries in children.

Therefore, the aim of the present study was to investigate individual determinants associated with traumatic dental injuries in schoolchildren 8 to 10 years of age.

## Methods

### Study design and sample selection

A cross-sectional study was conducted with 739 schoolchildren 8 to 10 years of age at public and private schools, after approval from the Human Research Ethics Committee (certificate number: 10514619.2.0000.5187). In a universe of 73 public schools and 58 private schools with 23,592 students [11], 349 students from 9 public schools and 390 students from 14 private schools were selected, ensuring sample representativeness through a randomization procedure executed in the Microsoft Office Excel 365 software.

Sampling weights were considered during the analysis (complex analysis). The sample was calculated using probabilistic sampling by clusters stratified in two stages (schools and children) proportional to the six administrative districts and schools (public and private) of the city. Simple random sampling was then performed within each school while maintaining the proportion of students within each administrative district. The number of students included in the study was proportional to that found at public and private schools in the population. Although more public schools are found in the city, many have a smaller number of students compared to some private schools that have a larger number of students. Thus, proportionally and after the simple random sampling at the selected schools, respectively, 349 and 390 students were obtained, corresponding to the same proportion found in the total population of the study.

The sample size was calculated for analytical studies of comparison between two independent proportions with the aid of the G* Power software program, version 3.1 (Franz Faul, Universitat Kiel, Germany), considering a 95% significance level and 5% acceptable rate of error. The estimates for the sample calculation were based on data from the pilot study regarding the proportion of TDIs in children with and without daily screen times of more than 2 h (10.7% and 21.2%, respectively), leading to a minimum sample of 380 children. A design effect of 1.6 was applied to increase the variation in the sample and 20% was then added to compensate for possible dropouts. The cluster sampling procedure was based on the literature considering two clusters (districts/geographic areas of the schools and schools). The calculation of the design effect was based on the variation in the data of the sample [12–14]. The final sample comprised 760 schoolchildren 8 to 10 years of age.

Children who wore orthodontic appliances, those with special physical, behavioral, or sensorial needs previously diagnosed by a specialist and those who parents/guardians had not lived in the same house with the children for at least 6 months were not included in the study.

### Training and calibration

Examiners underwent training and calibration exercises for the diagnosis of TDI conducted in two steps – theory and practice. In the first step, an expert in the field oriented the examiners regarding the diagnosis of TDI according to the criteria proposed by Andreasen [15] using images projected with Datashow. In the practical step, the four examiners diagnosed TDIs in 40 schoolchildren on two separate occasions with a seven-day interval. The Kappa statistic was used for the determination of intra-examiner agreement (*K* = 0.89 to 0.90) and inter-examiner agreement (*K* = 0.81 to 0.88).

#### Pilot study

A pilot study was conducted with 40 children from public and private schools to test the methods. The results of the pilot study revealed that the proposed methods were adequate. The children who participated in this step were not included in the main study.

### Individual determinants

Parents/guardians answered a sociodemographic questionnaire addressing the child’s gender, mother’s age (categorized by the median), guardian’s schooling (≤ 8 years or > 8 years of study), guardian’s marital status, the practice of sports at school, the use of electronic devices and the frequency of use of such devices (≤ 2 h or > 2 h per day) by children [16, 17] as well as questions addressing TDI (cause of injury and where the injury occurred). The children were asked how the injury occurred – whether it was a fall, collision, during the practice of sports, during a fight, or a traffic accident – and where the injury occurred – school, home, street, another place, or ‘does not remember’.

The parents/guardians also answered the Family Adaptability and Cohesion Evaluation Scale (FACES III) [7]. This instrument is used to collect data on family functioning and comprised 20 items (odd-numbered items address family cohesion and even-numbered items address family adaptability). FACES III has been translated and validated for use in Brazil [18]. Each item is scored from 1 (almost never) to 5 (almost always). The total is calculated by the sum of the items and ranges from 10 to 50 for each domain [19]. In the present study, only the questions addressing family adaptability were considered. Adaptability regards flexibility among the members of the family in terms of changes in power relations and is classified as rigid (10–19 points), structured (20–24 points), flexible (25–29 points), or chaotic (30–50 points).

### Clinical examination

The examinations were performed at the schools at predefined times in a separate room with the child in the sitting position. The examiners used personal protective equipment, a portable LED headlamp (Petzl Zoom headlamp, Petzl America, Clearfield, UT, USA), sterile mouth mirrors (PRISMA, São Paulo, SP, Brazil), sterile WHO probes (OMS-621-Trinity, Campo Mourão, PA, Brazil), and gauze to dry the teeth. The diagnosis of TDI followed the classification proposed by Andreasen [15]: absence of trauma, enamel fracture, enamel and dentine fracture, complicated crown fracture, extrusive luxation, lateral luxation, intrusive luxation, and avulsion. Discoloration, combined injuries and restorations due to TDI were also considered. Based on these clinical situations, TDI was dichotomized as present or absent. TDI was also classified as non-complicated (involving only the enamel and enamel/dentine) or complicated (other forms of TDI). Only the maxillary and mandibular incisors and canines were examined for TDI.

### Contextual determinants

The contextual variables of interest were the type of school (public or private) and physical structure of the school (type of courtyard type of courtyard [Hardfacing – cement/ceramic/granite or coating soft – sand/grass] and whether stairs had a handrail).

### Statistical analysis

Statistical analyses were performed with the aid of SPSS Statistics (SPSS for Windows, version 25.0, IBM Inc, Armonk, NY, USA). Descriptive statistics were first conducted with the calculation of absolute and relative frequencies for the characterization of the sample. The prevalence ratio (PR) compares the frequency of a condition across different groups, providing a measure of the association between an exposure and an outcome. It is calculated by dividing the prevalence of the condition of interest in the exposed group by the prevalence in the unexposed group. Unadjusted and adjusted multilevel Poisson regression models were run to describe associations between the independent variables and outcome. In the multilevel analysis, the null model was used as a comparative adjustment parameter. A model with only individual variables and another including individual and school variables were considered, in order to allow through deviance and variance partition coefficient (VPC) analysis, to compare the effect of the context under study on the prediction of the dependent variable, justifying the relevance of the contextual level in the analysis. The significance level was set at 5% (*p* < 0.05).

## Results

A total of 739 schoolchildren 8 to 10 years of age participated in the present study (response rate: 97.2%). In six schools, losses (*n* = 21) occurred due to three consecutive absences on the part of children (*n* = 16) and refusals to participate in the study (*n* = 5). Table 1 displays the absolute and relative frequencies of the variables analyzed. The prevalence of TDI was 16.2%. Most of the children had family adaptability classified as chaotic (69.6%), practiced sports at school (57.1%), and used electronic devices on a daily basis (61.0%), with screen time considered normal (≤ 2 h per day) (66.6%).

**Table 1:** Descriptive analysis considering traumatic dental injury and independent variables in children 8 to 10 years of age.

Individual variables	*N*	%
Traumatic dental injury		
Yes	120	16.2
No	619	83.8
Gender		
Male	367	49.7
Female	372	50.3
Child’s age		
8 years	269	36.4
9 years	240	32.5
10 years	230	31.1
Race		
White	255	34.6
Non-White	483	65.4
Mother’s age		
≤ 35 years	384	52.7
> 35 years	345	47.3
Guardian’s schooling		
≤ 8 years of study	317	43.0
> 8 years of study	420	57.0
Guardian’s marital status		
Single	285	38.6
Married	453	61.3
Practice of sports at school		
Yes	422	57.1
No	317	42.9
Daily use of electronic devices		
Yes	450	61.0
No	288	39.0
Screen time per day		
Excessive (> 2 h)	192	26.0
Normal (≤ 2 h)	492	66.6
Family adaptability		
Chaotic	514	69.6
Structured	225	30.4
**Variables related to traumatic tooth injury**		
Where injury occurred		
School	25	22.9
Home	22	20.2
Street	26	23.9
Does not remember	42	33.0
How injury occurred		
Fall	24	21.8
Accidental collision	30	27.3
Traffic accident	10	9.1
Does not remember	51	41.8
Classification of injury		
Non-complicated	112	99.6
Complicated	08	0.4
Number of teeth affected		
One	89	78.1
Two or more	31	21.9
**Contextual variables**	*N*	%
Type of school		
Public	349	47.2
Private	390	52.8
Type of courtyard		
cement/ceramic/granite	593	82.0
Sand/grass	130	18.0
Stairs with handrail		
Yes	604	81.7
No	135	18.3

A large portion of the TDIs occurred on the street (23.9%) and accidental collision was the most frequent main cause (27.3%). Nearly all TDIs were classified as non-complicated (99.6%), and only one tooth was affected in most cases (78.1%). Regarding contextual variables, most children studied at private schools (52.8%), with cement/ceramic/granite courtyards (82.0%) and stairs with handrails (81.7%).

Table 2 displays the variables that were considered control factors for inclusion in the statistical model (those with *p* < 0.20 in unadjusted analysis). The individual variables were guardian’s schooling, guardian’s marital status, the practice of sports at school, daily use of electronic device, screen time, and family adaptability. The contextual variables were the type of school and the type of courtyard surface.

**Table 2:** Unadjusted Poisson regression analysis for complex samples considering individual and contextual variables according to traumatic dental injury in children 8 to 10 years of age.

	Traumatic dental injury			
Variables	Yes	No	*p*	Unadjusted PR
	*n* (%)	*n* (%)		
Sex				
Male	62 (16.8)	308 (83.2)	0.70	1.02 (0.62–1.37)
Female	58 (15.7)	311 (84.3)		1
Mother’s age				
≤ 35 years	60 (16.1)	322 (83.9)	0.82	1.05 (0.70–1.55)
> 35 years	58 (16.8)	287 (83.2)		1
Guardian’s schooling				
≤ 8 years of study	60 (18.9)	258 (81.1)	0.09	1.39 (0.94–2.05)
> 8 years of study	60 (14.3)	359 (85.7)	-	1
Guardian’s marital status				
Single	61 (13.5)	392 (86.5)	< 0.01	1.67 (1.13–2.48)
Married	59 (20.7)	226 (79.3)	-	1
Practice of sports at school				
Yes	79 (18.7)	343 (81.3)	0.03	1.55 (1.03–2.33)
No	41 (12.9)	276 (87.1)	-	1
Daily use of electronic devices				
Yes	65 (22.6)	223 (77.4)	< 0.01	2.09 (1.41–3.10)
No	55 (12.2)	395 (87.8)	-	1
Screen time per day				
Excessive (> 2 h)	88 (17.9)	404 (82.1)	0.02	1.77 (1.06–2.94)
Normal (≤ 2 h)	21 (10.9)	171 (89.1)	-	1
Family adaptability				
Chaotic	98 (19.1)	416 (80.9)	< 0.01	2.17 (1.32–3.55)
Structured	22 (9.8)	203 (90.2)	-	1
**Contextual variables**				
Type of school				
Public	72 (20.6)	277 (79.4)	< 0.01	1.85 (1.24–2.75)
Private	48 (12.3)	342 (87.7)	-	1
Type of courtyard				
cement/ceramic/granite	105 (17.7)	488 (82.3)	0.03	1.93 (1.05–3.56)
sand/grass	13 (10.0)	117 (90.0)	-	1
Ramps/stairs with handrail				
No	23 (17.0)	112 (83.0)	0.78	1.27 (0.77–2.10)
Yes	97 (16.1)	507 (83.9)	-	1

Table 3 displays the results of the multilevel Poisson regression analysis. The individual factors associated with the outcome were single parents (PR = 2.33; 95% CI: 1.79–2.66), the practice of sports (PR = 2.46; 95% CI: 1.70–3.22), the daily use of electronic devices (PR = 1.78; 95% CI: 1.31–1.81), more than 2 h per day of screen time (PR = 3.84; 95% CI: 1.94–4.28), and belonging to a chaotic type family (PR = 4.22; 95% CI: 3.44–4.99). The contextual variables that remained associated with the outcome were attending a public school (PR = 1.77; 95% CI: 1.02–3.05) and rigid courtyard floor (cement/ceramic/granite) (PR = 1.99; 95% CI: 1.15–3.15).

**Table 3:** Multilevel analysis of individual and contextual variables associated with traumatic dental injury in children 8 to 10 years of age.

	Model 1 (‘null’)	Model 2		Model 3	
	PR (95% CI)	*p*	PR (95% CI)	*p*	PR (95% CI)
Intercept	0.29 (0.10–0.64)		1.64 (1.43–1.86)		6.16 (5.11–7.21)
**Individual variables**					
Sex					
Male	-	-	-	-	-
Female	-	-	-	-	-
Age					
8	-	-	-	-	-
9	-	-	-	-	-
10	-	-	-	-	-
Mother’s age					
≤ 35 years	-	-	-	-	-
> 35 years	-	-	-	-	-
Guardian’s schooling					
≤ 8 years of study	-	-	-	-	-
> 8 years of study	-	-	-	-	-
Guardian’s marital status					
Single	-	<0.01	1.90 (1.50–3.34)	0.03	2.33 (1.79–2.66)
Married	-	-	1	-	1
Practice of sports at school					
Yes	-	<0.01	3.68 (1.170–2.28)	0.01	2.46 (1.70–3.22)
No	-	-	1	-	1
Daily use of electronic devices					
Yes	-	0.03	2.38 (1.81–2.69)	<0.01	1.78 (1.31–1.81)
No	-	-	1	-	1
Screen time per day					
Excessive (> 2 h)	-	0.01	1.76 (1.71–2.56)	<0.01	3.84 (1.94–4.28)
Normal (≤ 2 h)	-	-	1	-	1
Family adaptability					
Chaotic	-	0.003	1.95 (1.26–3.01)	0.04	4.22 (3.44–4.99)
Structured	-	-	1	-	1
**Contextual variables**					
Type of school					
Public	-	-	-	0.03	1.77 (1.02–3.05)
Private	-	-	-	-	1
Type of courtyard Cement Sand	- -	- -	1 -	0.04 -	1.99 (1.15–3.15) 1
Ramps/stairs with handrail					
No	-	-	-	-	-
Yes	-	-	-	-	-
Deviance (-2loglikelihood)	40371.18	-	36624.19	-	29339.90

Values are presented as OR (95% CI). VPC, variance partition coefficient for random intercepts of mixed effects models. * Significance level at *p* < 0.05. ^a^*Unconditional model. ^b^*Individual covariates. ^c^*Subject and school-level covariates.

## Discussion

The conceptual hypothesis of the present study was confirmed. The prevalence of TDI was higher among children who used electronic devices and whose screen time was more than 2 h per day. The prevalence of TDI was also higher among children of single parents, those whose families were classified as chaotic, those who studied at public schools, and those whose schools had a rigid courtyard floor (cement/ceramic/granite). This is the first study to evaluate associations between TDI and both the use of electronic devices and excessive screen time in children 8 to 10 years of age. In the present investigation, being a child of a single parent, the daily use of electronic devices, spending more than 2 h a day in front of the screen of electronic devices, and having family adaptability classified as chaotic were individual determinants of TDI, whereas attending public school and a rigid courtyard floor (cement/ceramic/granite) at school were contextual determinants.

While technological advances have facilitated access to electronic devices, such devices also have negative impacts on overall health by promoting an increase in sedentary behaviour [20–22]. Moreover, greater screen time is associated with greater atypical sensory responsiveness [21] and less concentration [17], consequently increasing the likelihood of accidental injuries and TDI. Further studies are needed to gain a better understanding of this association, and pediatric dentists should be aware of the need to investigate associations between TDI and the use of electronic devices as well as the time spent in front of the screen of such devices.

Children who lived with single parents were more likely of having TDI compared to those who lived with married parents, which is in agreement with data described in the literature [22, 23] and may be the result of insufficient health care [24, 25]. Single parents have greater financial burdens and are often more occupied with their daily activities [24, 25]. This aspect is an important social determinant that should be considered in prevention measures and health education directed at TDI in children of single parents.

The family environment is an aspect that has recently been explored with regard to the occurrence of TDI. Besides family structure, power relations among family members can exert an influence on the needs of children [8]. In the present study, the chaotic type of family adaptability influenced the occurrence of TDI. This finding may be explained by the fact that chaotic families have greater flexibility, no established family leader, frequent changing of rules, and less supervision of daily activities [26]. This type of family behavior may predispose children to TDI. The study of the influence of family adaptability on the occurrence of TDI in children 8 to 10 years of age is unprecedented. This investigation underscores the need for novel approaches to prevention and health promotion focused on the family environment [27].

The prevalence of TDI was higher among students who practiced sports at school. Such activities are part of the curriculum of public and private schools and constitute an important risk factor for TDI [28, 29]. The high prevalence of TDI in this phase [1, 2] and the lack of encouragement regarding the use of mouthguards during the practice of sports [30] make this group of children more vulnerable to TDI. It is necessary to encourage the adoption of educational measures at schools for parents, teachers of sports modalities, and children regarding the importance of the use of mouthguards for the prevention of TDI. The present investigation contributes school-based findings on this association and can assist in the establishment of TDI prevention measures for schoolchildren in the mixed dentition phase.

Besides the learning-teaching experience, the school plays a fundamental role in the physical, emotional, and social well-being of students [31]. However, few studies have investigated the influence of the structural aspects of schools on the prevalence of TDI. In the present study, the contextual factors that remained associated with TDI were public school and the presence of rigid courtyard floors (cement/ceramic/granite). Type of school is an indication of socioeconomic status in Brazil [1, 32]. Previous studies reported that a lower socioeconomic status is associated with a greater occurrence of TDI [1, 31, 32]. Another point to consider is that children spend a large part of their time in the school setting, and its physical structure may exert an influence on the occurrence of TDI. Indeed, the presence of a rigid courtyard floor (cement/ceramic/granite) was a determinant of the greater occurrence of TDI in the present investigation, which is in agreement with data described in a previous study [10]. Most of the schools that participated in this study had rigid courtyard at school, which is not in line with the recommendation of soft surfaces in environments designed for children to prevent accidental injuries, including TDI [33, 34].

This study enabled the assessment of the association of individual and contextual determinants with the prevalence of TDI and is a representative, school-based study that included variables not previously explored in terms of the association with TDI. In the present investigation, data were collected on a single occasion, and it was not possible to know whether the child had been transferred to the current school only recently. Additionally, conducting radiographic assessments in a school-based study is not feasible and the clinical examination is limited to identifying clinical signs of past traumatic dental injuries.

The results are relevant for the planning of public policies directed at the prevention of accidents and unintentional injuries in the school setting. The findings suggest that greater attention should be given to the school environment not only in the form of health actions but also the creation of physical structures that promote safer schools.

## Disclosure statement

The authors report no conflicts of interest.
